# Early Crack Detection of Reinforced Concrete Structure Using Embedded Sensors

**DOI:** 10.3390/s19183879

**Published:** 2019-09-09

**Authors:** Joyraj Chakraborty, Andrzej Katunin, Piotr Klikowicz, Marek Salamak

**Affiliations:** 1 NeoStrain Sp. z o.o, Lipowa 3, 30-702 Krakow, Poland; 2 Institute of Fundamentals of Machinery Design, Silesian University of Technology, Konarskiego 18A, 44-100 Gliwice, Poland; 3 Department of Mechanics and Bridges, Silesian University of Technology, Akademicka 5, 44-100 Gliwice, Poland

**Keywords:** NDT, diffuse ultrasonic wave, damage detection, ROC, information fusion, SHM

## Abstract

The damage in reinforced concrete (RC) structures can be induced either by the dynamic or static load. The inspection technologies available today have difficulty in detecting slowly progressive, locally limited damage, especially in hard-to-reach areas in the superstructure. The four-point bending test on the benchmark RC structure was used as a test of the quality and sensitivity of the embedded sensors. It allowed assessment of whether any cracking and propagation that occurs with the embedded sensors can be detected. Various methods are used for the analysis of the ultrasonic signals. By determining the feature from the ultrasonic signals, the changes in the whole structure are evaluated. The structural degradation of the RC benchmark structure was tested using various non-destructive testing methods to obtain a comprehensive decision about structural condition. It is shown that the ultrasonic sensors can detect a crack with a probability of detection of 100%, also before it is visible by the naked eye and other techniques, even if the damage is not in the direct path of the ultrasonic wave. The obtained results confirmed that early crack detection is possible using the developed methodology based on embedded and external sensors and advanced signal processing.

## 1. Introduction

Reinforced concrete with steel bars, which can be considered to be a composite material, is widely used in civil engineering structures because of its high load-carrying capacity and low maintenance cost. In a reinforced concrete structure, rebars are protected from the outside by a few centimeters of concrete called coating (refer to Eurocode 2 EN 1992 (section 4)), a crucial protective layer for the service life. They meet the needs of mechanical strength thanks to the reinforcements and bonding with concrete. However, concrete is a material that shows changes over the period of operation. Thus, several factors, such as the loading of the structure, the environment, or the attacks sustained over time cause degradation of concrete material. Therefore, due to environmental and mechanical aggression, which lead to the penetrating the material leading to electrochemical reaction (corrosion) that eventually attack the steel, result in a material degradation [1]. This corrosion is a critical factor in the mechanical strength of the structure. To prevent corrosion, it is crucial to detect and characterize the cracks that may appear on the surface of the concrete as early as possible, and, in particular, to be able to estimate the sufficient depth and opening of these cracks. Therefore, monitoring of changes in the condition of an RC structure and detecting microcracks before they develop into macrocracks, and by timely intervention, could lead to a longer life of the structure. However, the degradation encountered in the concrete structures appears at different stages of the service life. In addition to, sometimes, destructive testing is not allowed to determine a quality of concrete (also taking sample is dangerous for structure). For this reason, there is an increased demand for more precise non-destructive testing (NDT) techniques and, at the same time, more flexible evaluation in the ability to detect the quality of the concrete.

The main interest of non-destructive testing (NDT) as a tool of auscultation is that it allows the carrying out of investigations (even repeated over time) without affecting the operations. The NDT are popular measuring methods, which is increasingly applied to the structures and structural parts in reinforced concrete as part of the maintenance and inspection [2]. Presently, NDT used in civil engineering structures, are easier to handle due to the continuous development of measuring technology and, taking into account the “correct” evaluation of the results, provide a reasonable basis for a reliable assessment of the actual condition of the structure. There are various NDT techniques have been used for decades, currently more than 70 types of standardized testing methods can be applicable to the evaluation of concrete structures, e.g., acoustic emission [3,4,5,6], infrared thermography [7,8,9], ground-penetrating radar [10,11,12,13], Fiber Bragg grating (FBG) [14,15], or digital image correlation (DIC) [16,17,18].

The traditional methods used for detecting cracks are mainly based on the measurement of global deformations. Unfortunately, the sensitivity and precision of this type of measurement are quite weak, the density of cracking must reach comparatively high values before its impacts start to be detectable in terms of deformations [19,20,21]. Acoustic emission (AE) can be a promising technique [8,10]. Listening to AE events gives information earlier than the visible opening of cracks, but the interpretation of results is always a difficult matter. This is because most AE events occur just before the propagation of microcracks [4]. The lack of significant AE activity at the initial stages of loading causes difficulty in distinguishing between background noise and acoustic events related to the crack. The techniques using ultrasonic waves velocity are particularly interesting because of the direct relationship between characteristics of wave propagation and the stage of damage to the material [22].

Ultrasonic Pulse Velocity (UPV) method is most commonly used to detect the quality of concrete, the position of crack or deepness inside both reinforced and masonry structures. In the ultrasonic inspection, the most widely used modes are longitudinal and shear waves for the propagation. Zhong and Yao et al. [23] identified the self-healing capability of normal and high-strength concrete damaged under compressive loads at several periods using UPV measurements. In [24], the application of ultrasound diffusion to detect damage in aluminum plates was used. The characterization of concrete by the propagation of ultrasonic waves is a usual way to evaluate the potential resistance of a structure. The use of proven techniques such as transmission, echo pulse or surface waves can identify areas with weaken mechanical characteristics, or even to detect the presence of cracks. Usually, these techniques applied from the component surface. Here, the contact between the material and the ultrasonic sensors is not always of equal, stable quality, as they are usually coupled with water or glycerin. However, it is important to note that many experimental and environmental parameters can influence measurements due to surface connection. One should be very careful about the constant coupling of ultrasonic sensors. Otherwise, the use of evaluation methods will evaluate the slightest changes in the signal caused by changes in the state of the component. The ultrasonic velocity—compressive strength correlations that are generally used can only be applied under specific circumstances. Therefore, Bundesanstalt für Materialforschung und -prüfung (BAM) developed a novel ultrasonic transducer, which can be permanently embedded into concrete structure [25]. It allows the constant coupling of the embedded ultrasonic sensors to the concrete and the embedding in deeper areas inside a tested structure. The sensors are also suitable for the permanent investigations of concrete structures with the pulse velocity method. Moreover, embedding provides the ability to monitor areas that are conventionally no longer accessible from the component surface. Since acquired signals from these embedded sensors need to be analyzed to characterize the concrete, it is essential to use primary signal processing methods and approaches, such as features and simple statistical measures.

Another commonly used technique for detection of cracks in structures (e.g., plate or rod) is the group of ultrasonic guided waves (Rayleigh and Lamb waves) approach. In this technique, where guided wave modes are preferred to obtain a clear response from damage via a single-mode. However, if the structure is made of heterogeneous and strongly scattering material like concrete, guided waves are difficult to interpret (methods are restricted to components). For these situations, diffuse ultrasonic waves can be created by an impulse excitation, allowing many reflections to occur and resulting in a similar diffuse wave in the structure [26]. The challenges linked with diffuse waves is the complexity of the waveforms, because it allows many modes, as the structure can support during the propagation (like a random walk).

Addressing these challenges and extracting damage/change information from complex diffuse waves have been the subjects of the vast number of studies. For example, in [26], investigated small cracks under environmental changes. In [27], presented the efficacy of the ultrasonic technique in discerning healing from its failure. In [28], studied real crack and influence on the diffusion parameters (degradation of the signal scattered from structural deformation). The diagnosis of large cracks/notches and the monitoring of crack propagation using diffuse ultrasonic wave can be found in [28,29]. In [30], Michaels and Michaels have presented the structural change in a simple aluminum specimen using short-time cross-correlation of two diffuse ultrasonic signals recorded from the same transmitter and receiver, before and after damage. Anugonda et al. [31] investigate the propagation and scattering of ultrasound in concrete structure and determined the diffusion parameters. In [32], Won presented the measurement of the artificial cracks varying depth in the concrete specimens with diffuse ultrasound. Eunjong et al. [33] examined the water permeability and chloride ion penetrability of cracked concrete sample using diffuse ultrasonic signal and shown that the relations between crack width, water flow, and diffuse ultrasound parameters. Considering this, the diagnosis of propagation of microcracks in reinforcement concrete remains a significant challenge for NDT techniques, despite the special interest in making such degradation since these cracks may lead to undesirable premature failure. Advanced signal processing techniques, such as time-frequency domain analysis, statistical, matching pursuit, and other, could be useful for determination of damage-sensitive features. In most of the cases, different NDT techniques produce multiple decisions, often conflicting about the integrity of the monitored structure. In [34], the distributed fiber optic and coda wave techniques for damage investigation in concrete structure are presented, and they showed that both techniques achieved earlier damage detection than standard sensors. However, no statistical methods have been used to compare all the techniques. The above challenge led researchers to use fusion techniques at different levels of data processing. Information-level fusion has been used after data transferred into abstractions. In NDT techniques, distracted decisions at a high level of abstractions may be produced by several techniques about the integrity of the structure/material [35]. Typically, decision fusion is applied at the final stage of the process of evaluation. Ideally, decision fusion reduces the level of uncertainty in the decision made by different techniques and produce more trusted decisions with high level of confidence.

Cracks in rebar-reinforced concrete beams provide a very useful first warning for the monitoring of structures in risk environments. In this paper, the cracks are caused by static load application on a reinforced concrete beam equipped with four embedded ultrasonic sensors in a four-point arrangement. On the transmitted ultrasonic signals, the different features are extracted as a function of the load. To evaluate the accuracy of the crack detection with the embedded ultrasonic sensors, the state change of the beam is monitored with additional NDT methods. Moreover, the implementation of decision fusion method may significantly reduce the level of uncertainty and enhance damage detection ability.

As it can be concluded from above literature review, the information fusion on various levels is a very helpful technique, which can provide earlier crack detection in undamaged structures due to the appropriately constructed decision-making algorithm. Moreover, for successful detection of damage one need to apply the processing algorithms with enough sensitivity to detect early cracks in a structure. Finally, the appropriate threshold should be established to distinguish between healthy and damaged structure and minimize the possibility of false damage indications. In this paper, the authors combined all these approaches in order to develop a damage detection system based on embedded sensors, which is sensitive to microcracks and robust to false damage indications, simultaneously. The originality of this paper covers the application of newly developed features based on advanced signal processing measures and functions, selection of the most sensitive ones to cracks, and their implementation into the fusion algorithm, which resulted in increasing of overall sensitivity to damage in considered benchmark structure.

The rest of the paper is organized as follows. In Section 1, the test object and experimental setups are described, then the acquired signal is explained along with proposed features. In Section 2, these features are evaluated with the different NDT techniques. The role of information fusion method is introduced, and the comparison of different NDT techniques is highlighted using receiver operating characteristic (ROC) curves also in Section 2. The whole paper is summarized and remarked in the end of Section 3.

## 2. Test Specimen and Methods

### 2.1. Test Specimen

**Figure 1:**
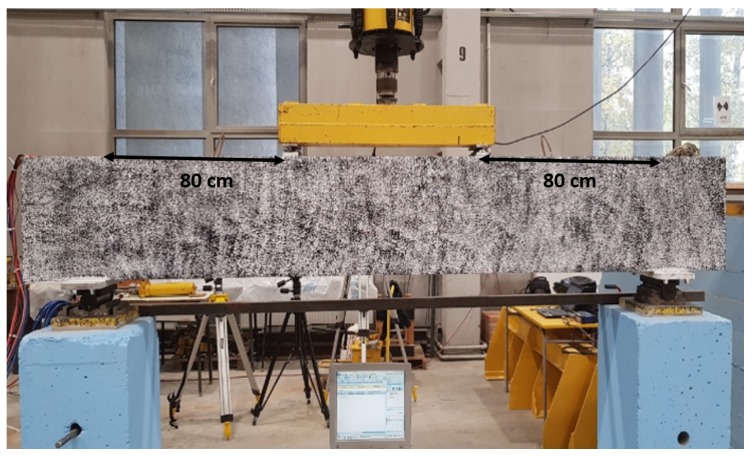
Measuring stand and beam load position.

The reinforced concrete (RC) beam was casted as a specimen for the following induced cracks propagation. Figure 1 shows the test specimen of the RC beam that has the following dimensions: 2.9 m × 0.4 m × 0.2 m (length × height × width). The primary reinforcement of the 10 mm diameter consists of three lower bars in the tensile zone and two bars in the corners of the compression zone. Transverse reinforcement consists of two-legged stirrups with a diameter of 6 mm arranged in the support zone at a spacing of 150 mm. The beam was made of concrete class C25/30 with compressive strength $F_{cd}=27.57$ MPa. The capacity of the beam was calculated according to Eurocode 2 EN 1992-1-1. The calculation results were used to determine the maximum breaking force of the beam.

**Figure 2:**
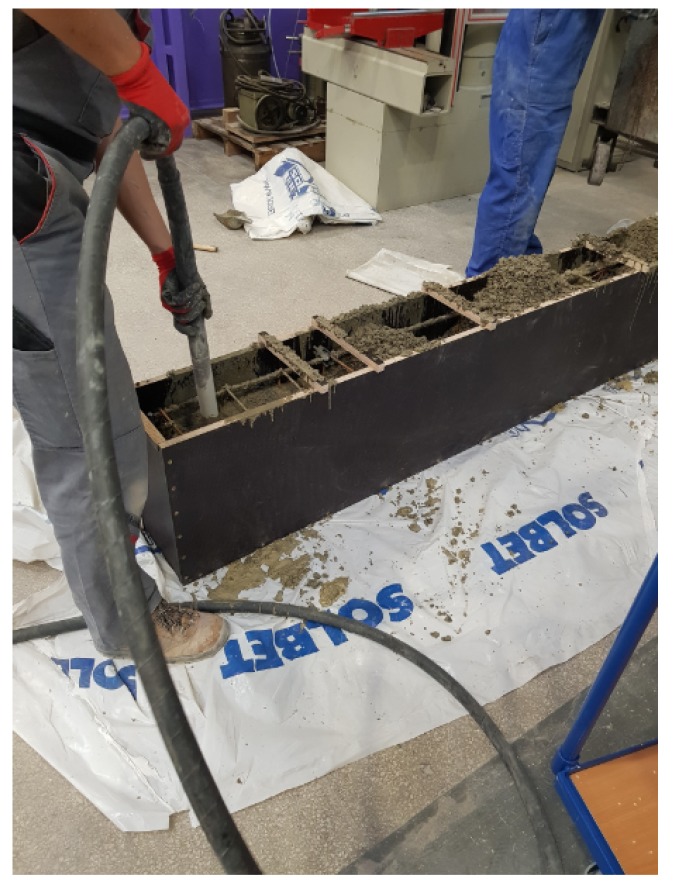
Vibration during pouring the concrete and collecting of specimens.

The reinforcement skeleton together with attached sensors was placed inside the formwork while maintaining the appropriate buffer cover. During laying, the concrete was properly compacted using vibrators (Figure 2).

**Figure 3:**
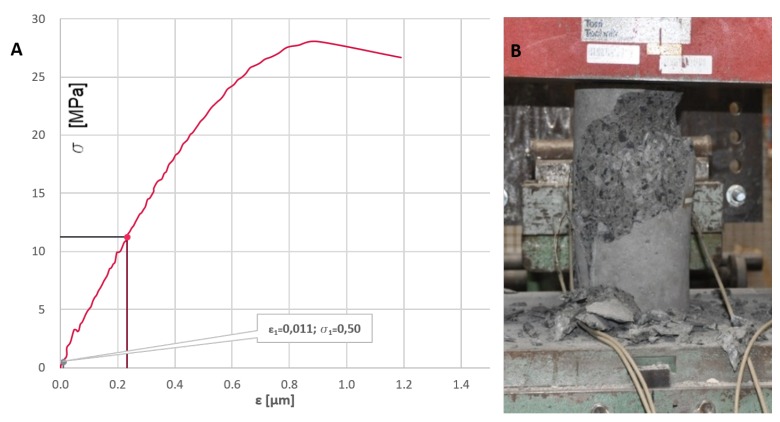
The σ - ϵ curve (**A**), and the view of the failed specimen after compression testing (**B**).

To evaluate the actual class of concrete and its mechanical properties, during the concreting, 12 additional specimens were manufactured in the form of cylinders with a diameter of 150 mm and a height of 300 mm following EN 12390-1 code. Both the beam and specimens were seasoned in similar conditions for 28 days. The beam was left in the lab after casting, but the temperature and moisture were close to constant. It was all the time indoor. The benchmark RC structure for elastic modulus were cured in water in constant temperature about 15 ${}^{\circ }$C. During the period of concrete hardening and seasoning, the measurements were started. The recorded signal from the internal sensors was sampled at intervals of 5 min. In this way, the influence of concrete bonding temperature and rheological phenomena, such as shrinkage and creep of concrete, was assessed. Specimens were tested after 7, 14, and 28 days, which allowed to evaluate the increase in strength over time and determine the actual class of concrete with its compressive strength and modulus of elasticity. During compression and testing until failure the concrete specimens after the mentioned duration of the casting works, the σ - ε curve and the elastic modulus were determined. The exemplary view of the σ - ε curve and the resulting tested specimen are presented in Figure 3.

**Figure 4:**
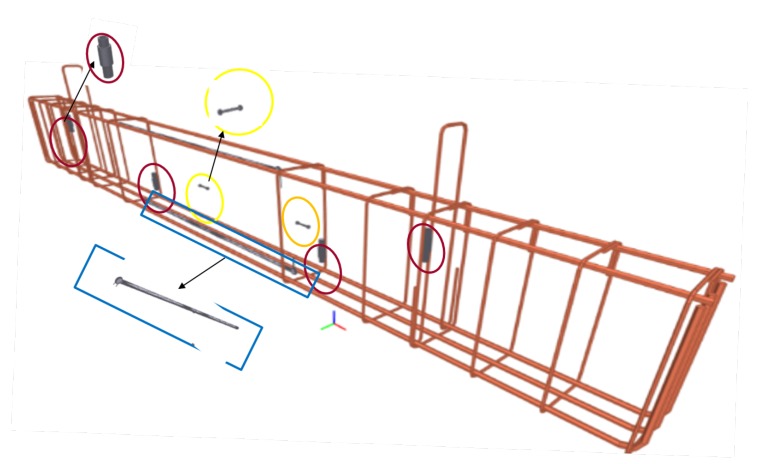
3D view of the beam reinforcement with sensors location.

**Figure 5:**
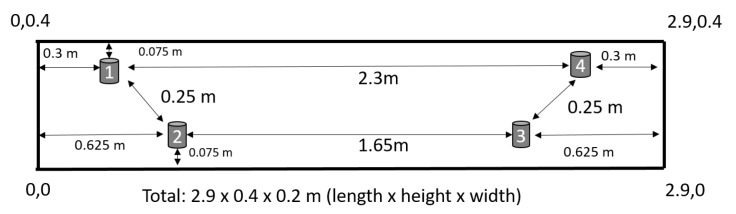
Ultrasonic sensor position.

During the preparation of the benchmark RC beam, the sensors were concreted. The location of concreted sensors together with reinforcement is shown in Figure 4. Four ultrasonic sensors (red box) especially developed for RC monitoring purposes (see [25] for more details) were attached on four vertical stirrups. The positions of ultrasonic sensors are shown in Figure 5.

The beam was also instrumented with two vibrating wire strain gauge sensors Ace Instrument${}^{®}$—model 1220 (green box), and two rebar stress meters Ace Instrument${}^{®}$—model 1260 (attached to the top and bottom of the rebar) were embedded inside the concrete (Figure 4). External linear variable differential transducer (LVDT) displacement sensor PLETRON${}^{®}$—model PSX 100 and two strain gauges were used to monitor deflection. DIC device was used to measure deflections with cracks propagation and width.

### 2.2. Data Acquisition System and Loading Schedule

After placing the beam on the measuring stand, the acquisition system and power supply were connected for initial readings. In the middle of the beam specimen, an external LVDT sensor was installed to measure the deflection, and strain gauges were glued to the upper and lower surfaces of the beam. The front wall of the beam was painted with markers that allows measuring the deformation with the DIC method. In the middle, the hydraulic jack and a load cell ZR DIORA${}^{®}$—model 25 set to transfer two-point force (Figure 1) were mounted. Then, the whole system was tested, and the control data recording was performed.

**Figure 6:**
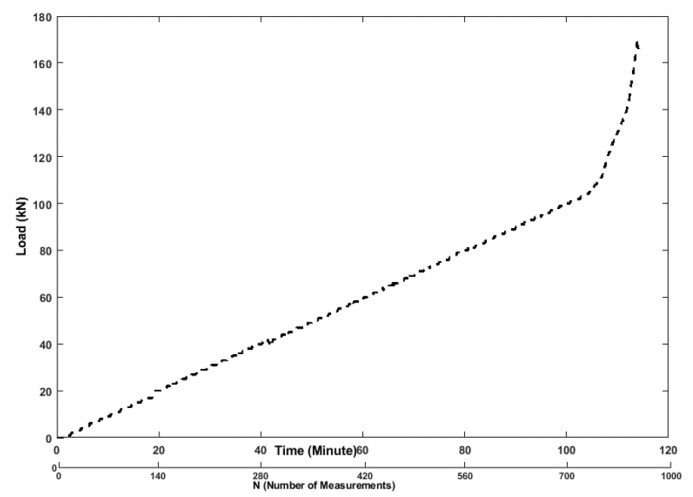
Loading schedule vs. number of ultrasonic measurements.

The loading machine is controlled by an analog controller. Since the main goal of the test was to evaluate the cracks evolution and the damage level with the increasing load using ultrasonic techniques (see Figure 6), it was decided to measure also the force. The loading rate was fixed at the beginning on 1 kN/min till 108 kN and then increased to 5 kN/min (Figure 6), which introduced appropriate stress/strain state in the tested specimen.

**Figure 7:**
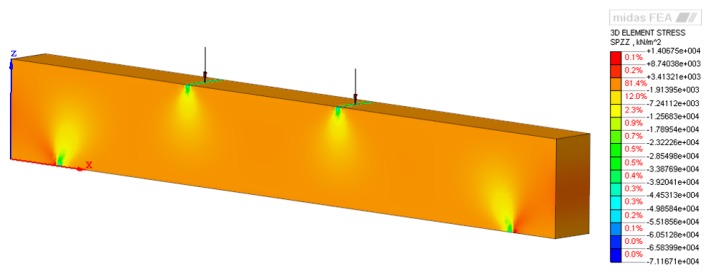
The strain distribution (Midas).

To visualize the strain distribution, the preliminary numerical FE calculations were performed basing on the material properties resulted from the quasi-static compression tests (see Section 2.1). For the purpose of examination of strain distribution, the static non-linear analysis was defined in Midas FEA, and the Newton-Raphson iteration procedure was used for FE calculations. The resulting strain distribution of the tested structure was presented in Figure 7. The force and the LVDT outputs were recorded on a computer using NI DAQ card data logger system, respectively HBM${}^{®}$—model QuantumX MX1615B card for the displacement and GOM Correlate for the DIC measurement.

**Figure 8:**
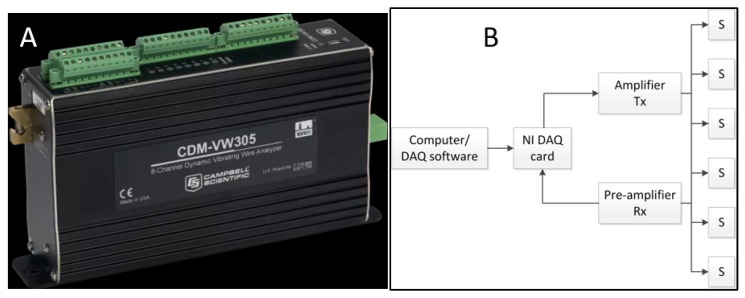
(**A**) Campbell Scientific data logger. (**B**) Data acquisition block diagram of the ultrasonic system.

The data is collected by connecting the computer directly to the Campbell${}^{®}$ data logger for embedded traditional sensors and customized data acquisition system for ultrasonic sensors [25]. The data logger has 16 channels (see Figure 8).

The acquisition from vibrating wire strain gauges was made automatically in every 200 ms and stored in the computer hard disk drive. For ultrasonic measurement, the data acquisition system contains multi-channel data acquisition module which is connected with the amplifier and the pre-amplifier to amplify the input, and output then filter the signals (see Figure 8). In the laptop, custom software is installed to configure and control the measurement system and store the signals. The acquisition of the ultrasonic signal was made seven times a minute and stored in the laptop. The center frequency of the measured ultrasonic signal was around 60 kHz, considering proper resolutions for acquired signals, a sampling frequency of 1 MHz was used. The recorded duration was 5 ms for each measurement, allowing many reflections to occur and resulting in a similar diffuse wave.

Ultrasonic wave velocity and attenuation is increased by initiating cracks and can indicate as damage index [36,37]. Therefore, attenuation and velocity changes are almost linear with initiating cracks. However, the relation of stress and strain in not linear for concrete under different loading. The speed of elastic ultrasonic waves stretching through the solid based on the mechanical stress of the body [38]. Still, from the previous experimental studies [37,39,40,41], the observations and result presented almost linear phase under different load variation .

As the test was done in the laboratory in room temperature during winter, so it followed low temperature variations of ±0.5${}^{\circ }$C during the test. However, ultrasonic sensors were embedded inside the benchmark concrete structure, so it is less influenced by near surface changes [42]. They are therefore ignored in this work.

### 2.3. Methodology

**Figure 9:**
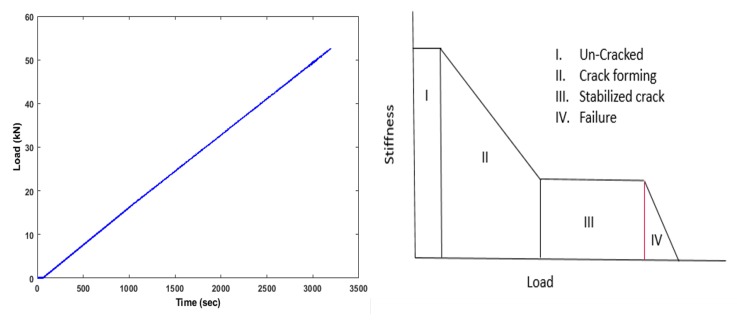
Generalized bending stiffness development where four phases are shown.

The basic idea of evaluating the bending stiffness of the RC beam under increasing in time vertical load is a deflection measurement used as a performance indicator (PI). The typical changes of beam bending stiffness during loading are illustrated in Figure 9. The degradation phase can be divided into four stages: (I) Un-cracked, (II) Cracks forming, (III) Stabilized cracks and, finally, (IV) Failure stage. Failure is typically yielding of steel reinforcement or crushing of concrete. During the test, the load was slowly and proportionally increased over time using a hydraulic jack compression machine. Simultaneously with the measurement of the load force, the responses from all sensors and measuring devices are registered.

**Figure 10:**
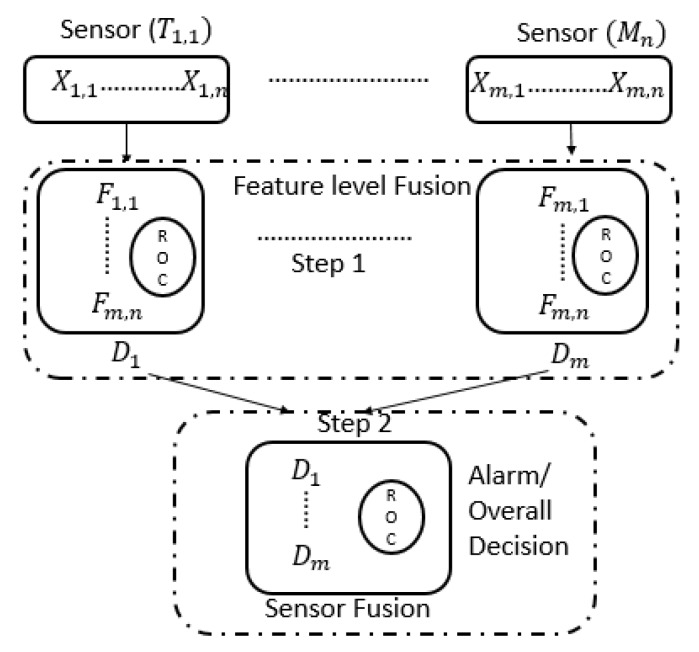
Two-step feature-based sensor fusion model.

As a first step (Figure 10), the feature level of fusion will apply to a signal coming from multiple sensors. The general frame of data fusion is investigated, for example: $X_{1,1}$,…, where $X_{1,n}$ is the vector of data from one ultrasonic transducer pair $T_{11}$ (one emitter and one receiver) and $F_{1,1}$ is a feature value from one transducer pair. In step 1, feature-level fusion represents the step of computing the features from all the sensors or sensors pair. In step 2, the decisions taken from ultrasonic sensor pair and outputs for other types of installed sensors, are fused using binary declaration in terms of operational changes (like presence/absence of load, presence/absence of crack/multiple cracks etc.).

### 2.4. Feature Extraction from Ultrasonic Signals

The feature extraction procedure involves the extraction of load/crack sensitive features from the data collected during the data acquisition periods to diagnosis the structural changes. The feature extraction process is done by using signal processing techniques to sensor data. There are different signal processing methods for the evaluation of ultrasonic time-domain signals. One of the signal processing procedures to obtain the feature from a measured ultrasonic time-domain signal is as follows: AR model residual error is the difference between the value from the measured signal and the predicted value from the baseline signal. This AR model residual error can be used as a changes/damage feature indicator. The next method is the decorrelation coefficient, which is used to compare the similarity of a signal with a reference undamaged signal. Next, the continuous wavelet transform (CWT), which is an effective signal processing approach, can be used as a changes/damage feature indicator. Wavelet transform is an estimator used to quantify the energy diffusion value from the acquired signal. The degradation of the material can also be determined by the difference of the peak amplitude in each window template of the time-domain signal for various change levels normalized by the reference undamaged condition. Finally, the short-time Fourier transform (STFT) is capable of providing information about signal behavior by performing a time-frequency analysis, from which it is possible to determine the time instants at which specific harmonics are present in the signal, as well as the power spectrum analysis. More information on feature extraction methods used in this study is available in [30,37,43,44,45,46]. The above-described features are summarized in Table 1.

### 2.5. Information Fusion

The idea behind the information fusion is to compare the information from multiple sensors to improve overall decision/localization. Data fusion can be classified into three levels: signal-level, feature-level, and decision/information-level fusion [47]. Signal-level fusion is called the lowest level fusion, which combines the raw signals from multiple sensors and, consequently, the new raw signal that is anticipated to be more informative. Feature-level fusion is also known as medium-level fusion, which involves calculating feature values from each sensor pair individually or compare the features from different sensors so that the most relevant ones to make a decision. Decision-level fusion is called the ultimate-level fusion in this hierarchy. In this level, each sensor can provide an independent decision based on its own features, and the results from all the features are then compared to get overall decision/localization.

In this paper, a voting scheme at the decision level is used for information-based fusion. The procedure of information fusion is illustrated in Figure 10. The first step is to select a threshold for each feature from the pair of ultrasonic signals using a voting scheme. This threshold was used to discriminate between the undamaged and damaged states in the features. Generally, the weight of the value for undamaged state is lower than loading or damaged state. The next is to use the receiver operating characteristic (ROC) to compare the features for overall decision [48]. The ROC curve is a metric used for statistical evaluation of a feature, and can be used to visualize the overall decision. In the voting scheme, an overall decision is made to differentiate between damage and undamaged class following to the maximum value of the voting index [49]:

(1) $$O_{d}=max\left(\sum_{n=1}^{N_{n}}W_{n}F_{n}\right),$$

where $N_{n}$ is the length of the vector of features used to assess the *n*-th sensor; $F_{n}$ is the feature value by the *n*-th sensors in relation to the different location; $W_{n}$ represents the voting weight (the default: $W_{n}=1$). The highest weighted average probability indicates a high possibility of the existence of damage.

### 2.6. Digital Image Correlation

Recently, DIC has developed as a stable and reliable tool for fracture or damage measurements. DIC detects the smallest deformations caused by stress on the surface of the examination subject. This pattern was used first as a reference. Later, after the application of loading, it was used as a pattern for comparison of similarity and determination the crack locations. The surface deformation of the benchmark RC beam was reflected by the shifts between pixels within these areas. To analyze this shifting different correlation algorithms can be used. This technique can measure deformations in the range of micrometers depending on the used camera and its distance to the surface of the beam. In the performed experiments the ARAMIS SRX DIC camera with a resolution of 4096×3068 pixels and GOM correlate system was used. The frame rate during testing was of 0.25 Hz.

## 3. Test Results and Discussion

**Figure 11:**
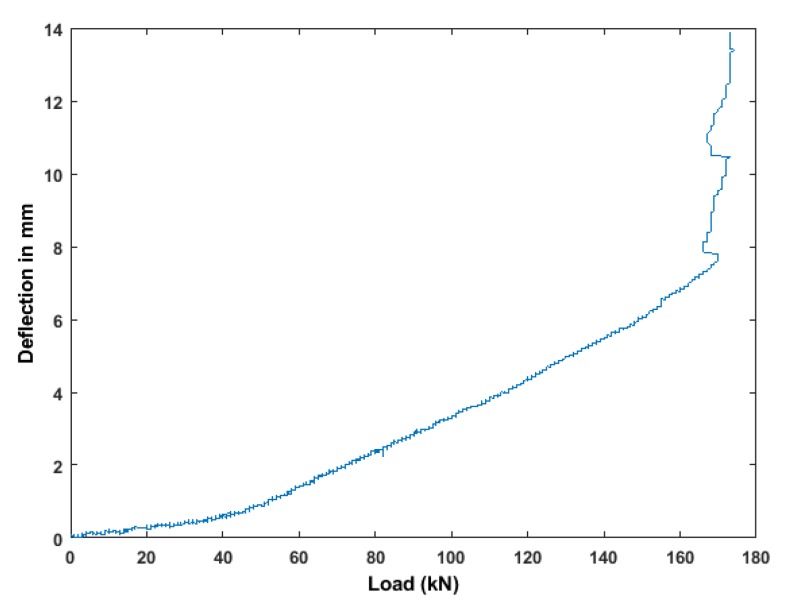
Deflection (LVDT) and load.

The crack opening displacement was measured by the vertical LVDT. It can be observed that there is a lack of symmetry in the loading or the shape of the beam. However, the variation of the crack width is proportional, meaning that one can suppose that the distribution of the crack opening displacement is linear all along the width of the beam. The measured load-displacement curve of the control beam is shown below in Figure 11, along with the load-displacement curve from DIC measurements can be seen in Figure 19B. It can be seen that cracking occurred at approximately 42 kN.

### 3.1. Flexural Performance

Four-point bending tests were performed on the specimen, and the conventional measurements of crack openings were obtained by using vertically placed LVDT. The ultrasonic analysis signals, obtained during the test, were processed to extract features explained above.

### 3.2. Analysis of Ultrasonic Features

To represent the effectiveness of the features mentioned in Section 2.4, all the features are computed from the time-domain signals collected from three pairs of ultrasonic sensors on the benchmark RC structure during the experiment depicted in Figure 1. In this paper, one pair of ultrasonic sensors was analyzed to investigate the cracks without being dependent on the location of the structure. Therefore, the ultrasonic sensors pair located in the top was chosen to investigate the maximum area of the benchmark structure (distance of 2.3 m). The interpretation of all the features explicitly incorporates comparison to a reference signal, and if there are not a certain amount of changes in amplitude, phase, and frequency of the signal is compared to the reference signal, and the condition of the structure is stated as a good health condition.

**Figure 12:**
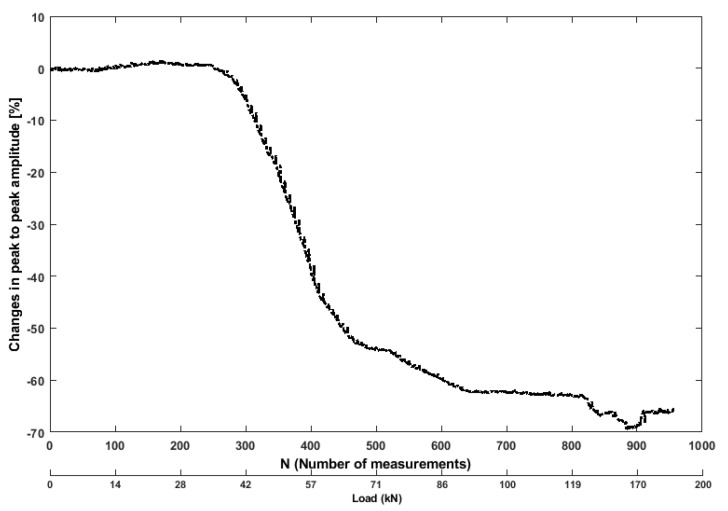
Values of Peak to peak amplitude feature from ultrasonic pair S01R04 time histories.

Figure 12 shows the exemplary plot for the features of the time-domain signal, i.e., peak to peak amplitude. The normalized error graphs are derived as a difference between the peak amplitude in each fixed-length time window (when the beam was subjected to external stress) and the reference of the undamaged state. Higher deviations were caused whenever the amplitude of time signals dropped with the external load increases and the progressive stage of damage (cracking). From Figure 12, it can be observed that the peak to peak amplitude changes as the stress level varies due to tension region (bottom) and compression region of the beam (top). The changes in the peak to peak amplitude stay within the range of 0–1.9%, when 0–35 kN (N=242 ultrasonic measurements, which is equal to 35 kN) of the load is applied. When the applied stress increases, the peak amplitude has an obvious drop, as signal is started to attenuate with less energy and a decrease of amplitude, which indicates the crack opening. The changes had an increasing trend when 35–90 kN of the load is applied, due to forming multiple cracks opening and propagation along the beam. However, when the load between 90–160 kN was applied, the changes remain stable at the brittle-ductile transition stage. Although the beam has a large deformation indeed, there were not enough new cracks in the specimen to obstruct the ultrasonic wave propagation. The reason for this stable propagation is that the reinforcement of the tested specimen carried the highest stress under such loading conditions.

**Figure 13:**
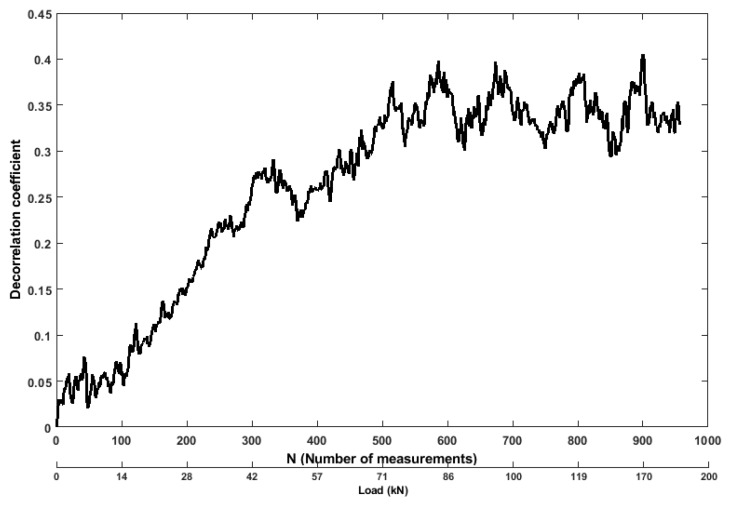
Values of decorrelation coefficient feature from ultrasonic pair S01R04 time histories.

The decorrelation coefficient is obtained in the normalized form, and it can be defined as the changes in the RC benchmark structure. The initial increase of this coefficient is originated by the bending of the benchmark specimen under increasing load, because it effects on the ultrasonic wave path. With a bending tensile force of 42 kN, the coefficient (Figure 13) increased by 0.27, compared to the reference state (before the start of the experiment), and sudden drop to 0.23 took place, which is indicating the development of microcracks in the structure. When the applied stress gets more significant (the results from a bending tensile force between 42 kN to 160 kN), and the phases of the signals shifted by more than 90${}^{\circ }$, which was caused by the propagation of the crack, whereby the decorrelation coefficient increased to 0.4. Decorrelation coefficient provides an appropriate value for judging about the rate of changes in measured signal comparing to the reference signal through the correlation analysis and directing of a particular relationship between the two signals. Therefore, it is showing that the signal analysis based on the decorrelation coefficient is more sensitive. Due to its sensitivity for small changes, decorrelation coefficient can give misleading information about the signal changes due to environmental effect.

**Figure 14:**
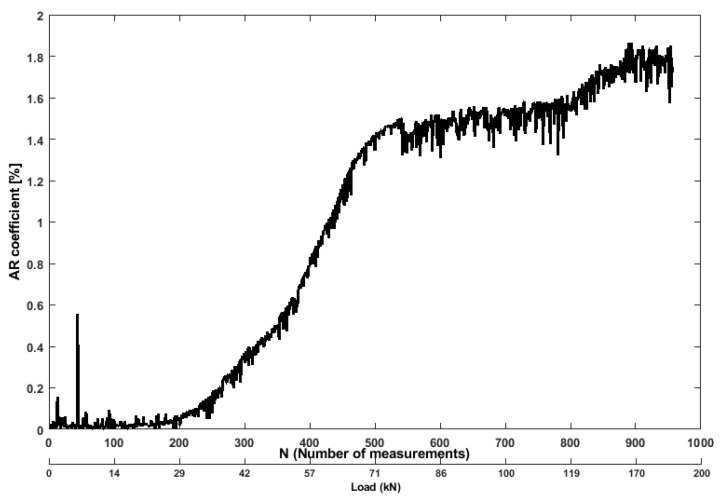
Values of AR residual error feature from ultrasonic pair S01R04 time histories.

Figure 14 indicates that increment of the level of nonlinearities (cracking) in the beam subjected to external stress, and simultaneously, the amplitude of the AR parameters (increasing the residual error) tends to decrease. The AR parameters were calculated by fitting the AR model to a baseline signal from one transducer pair (top) before the start of the experiment using the RMSE technique (see [43] for more details). If cracks are present in the structure, the residual errors will increase due to higher attenuation (decreasing the AR parameters amplitude), which is caused by the increased crack width under loading. From Figure 14, it can be observed that the residual error increases as the stress level increases in the beam. The results from a bending tensile force between 36 kN to 160 kN, the rate of AR residual error increases up to 1.9 % due to energy attenuation, which indicates the opening and propagation of cracks.

**Figure 15:**
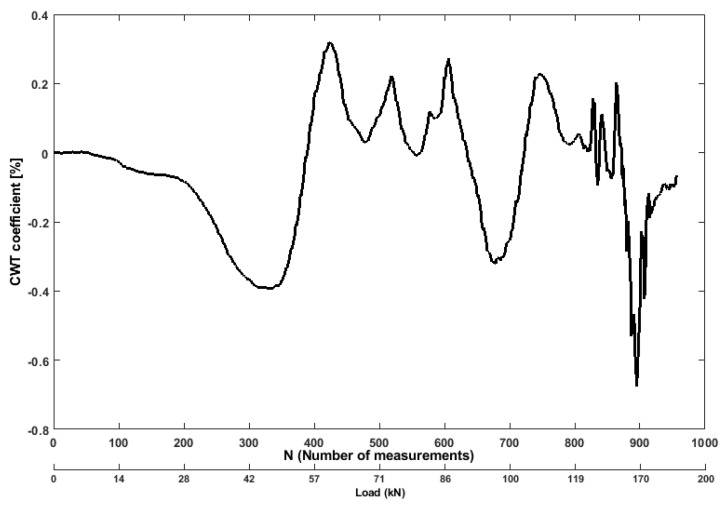
Values of CWT coefficient feature from ultrasonic pair S01R04 time histories.

The plot for Continuous wavelet transform (CWT) is depicted in Figure 15. The energy of a signal is derived from the CWT transform from the raw signal time-series. The extracted feature from this time-frequency domain analysis is more useful than time domain only. An energy vector is established by computing the energy of each branch (scalogram) to show the energy distribution towards the frequency bands. From the comparison of CWT coefficients between the changed/damaged state and reference undamaged state (as shown in Figure 15), a noticeable deviation can be found in different frequency bands. The proposed CWT energy coefficient is obtained by calculating the root-mean-square deviation in percentage between the energy vector of the health state and that of the damaged state. From Figure 15, it can be observed that the coefficient decreases as the bending tensile as the level increases between 36 kN to 48 kN in the beam, and then it dramatically increases as the load increases between 49 kN to 60 kN. This is because most of the cracks appear on the concrete surface parallel to the load application under compression. Ultrasonic wave propagation through such direction, therefore, may miss encountering cracks. There are no large values of the wavelet coefficients since the specimen has been destroyed by the horizontal splitting cracks that prevented the propagation of ultrasonic waves from transducer 1 to transducer 2 through the concrete.

**Figure 16:**
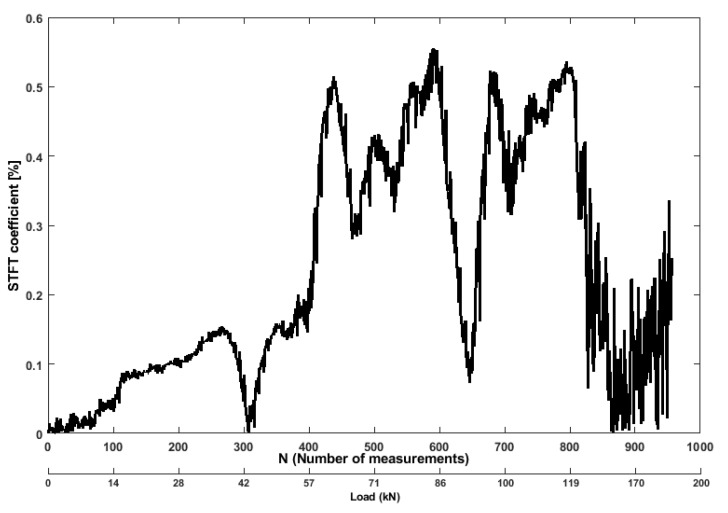
Values of STFT coefficient feature from ultrasonic pair S01R04 time histories

The STFT, conventional as a spectrogram, corresponds to the energy distribution of a raw signal. It can be defined the amount of energy contained in the diffuse signal as a damage-sensitive feature. The initial increase of this coefficient is originated by the bending of the benchmark specimen under increasing load, related to the amount of energy released by the specimen. With a bending tensile force of 38 kN, the coefficient (Figure 16) increased by 0.14, compared to the reference undamaged state, and sudden drop to 0.002 took place, which is indicating the signal strength decreased due to development of microcracks in the structure. It can be observed that after the load of 115 kN was applied, the strength of the signal gradually decreased as multiple cracks started to propagate through the surface, until the last phase of loading.

### 3.3. Analysis of Embedded Strain Gauges

**Figure 17:**
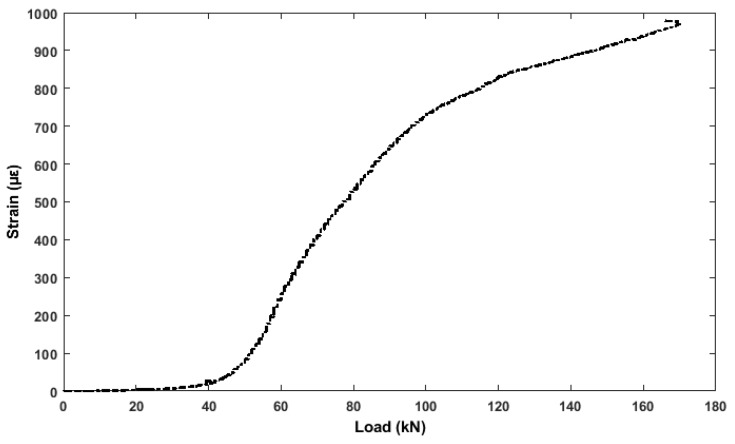
Strain (bottom vibrating wire) vs. Load.

The vibrating wire strain gauges embedded inside the concrete structure (in the middle of the beam), register a steady increase in strain from 1 up to 1000 με, during loading (Figure 17). The increase is caused by the elastic bending of the beam. The growing strain in the bending tensile state from 43 με onwards, indicates an inelastic change as the surface cracks.

**Figure 18:**
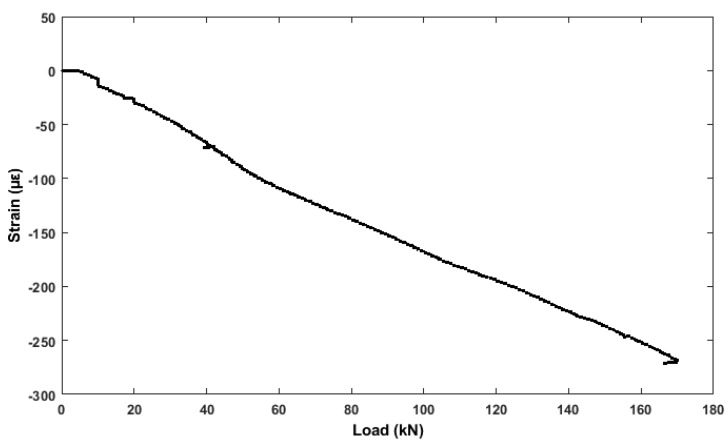
Strain (attached with top rebar) vs. Load.

Figure 18 indicates that the rebar strain gauges embedded with top of the rebar in the RC benchmark structure, register a steady increase in strain from 1 up to 272 με, during loading indicates an elastic change.

### 3.4. Analysis of DIC

**Figure 19:**
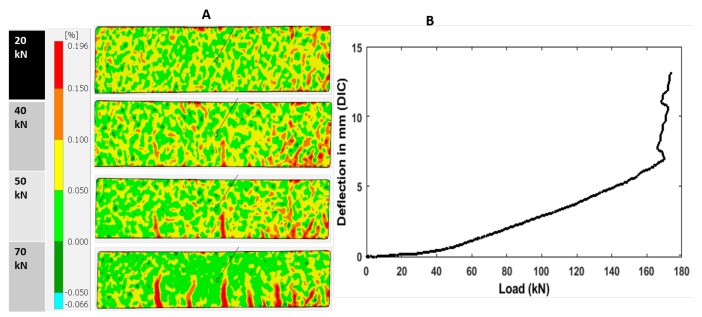
Crack propagation along with strain map (**A**) and Deflection (**B**) at different load levels of specimen (1st stages).

**Figure 20:**
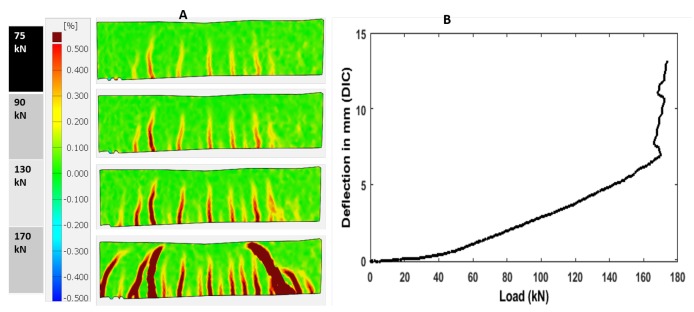
Crack propagation along with strain map (**A**) and Deflection (**B**) at different load levels of specimen (2nd stages).

To investigate the ability of DIC to detect the degradation and characterization of the material, the data obtained from DIC frames were matched with corresponding force—deformation and beam depth—crack width profiles. The displacement of the benchmark RC beam at each load step is determined by locating each subset from the baseline image (pixel subset) in an image of the deformed test specimen through the use of the highest correlation to the reference subset. Figure 19 and Figure 20 show the state of cracks along with strain fields at selected levels of load (A) and displacement curve (B). These figures are essential for evaluation of the state of deformation and understanding the cracking behavior of the material, especially when specimen with different properties were compared. It can be observed from Figure 19 for the benchmark RC beam that the highest level of strains is observed in the first stages (0–50 kN) of loading, and distributed through the maximum moment region immediately after peak stress and strain localization became to be recognizable as is represented by red color. In the end, one small crack is observed at 40 kN. Then one major crack together with several other cracks (Figure 19) was visible. The first signs of deformation are at one bending tensile strength of 42 kN, which correlates with the results of embedded strain gauges and displacement sensor. When the specimen surface was examined for cracks, these cracks were not visible by the naked eye. However, through the DIC, the propagation of deformations upward in the direction of load entry is visible. At bending tensile strength of 52 kN, from this period, forming cracks became visible by naked eye. However, in the second stages (55–170 kN), DIC represents that more cracks form in the specimen, which are not visible by the naked eye. At 80 kN, the cracks are started to be visible with the naked eye. Figure 20 shows the increasing propagation of the deformation in the direction of the load, which correlates well with all the sensors.

### 3.5. Features Comparison Using ROC Curve

To validate the feature-level fusion, five ultrasonic features were computed from the data collected on the benchmark RC specimen. The decision and creditability of these features was analyzed using ROC curves. The threshold was defined from the voting scheme and swept over the range of the feature values, and the probability of detection (POD) is plotted versus the false alarm rate (FAR). The ROC curve is a perfect detector which measures the value area under the curve (AUC). The accurate classifier corresponds to the maximum AUC (maximum AUC = 1). Therefore, larger AUC values indicate better performance. In the next stage of the algorithm (see Figure 10), features from different sensors are analyzed using a voting index threshold for ROC curves. In the end, the final decision was evaluated.

**Figure 21:**
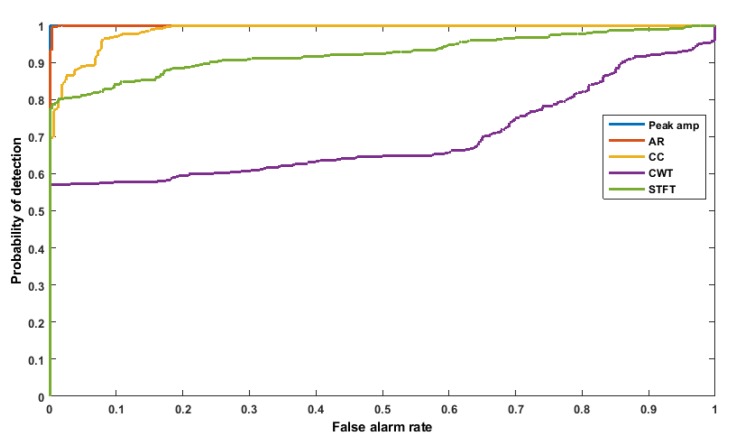
ROC curves for all the features in RC beam.

The proposed features from the ultrasonic signal are compared for sensor pair 01–04 in Figure 21 via their ROC curves. It can be seen that all the features perform fairly well in their ability to detect crack opening and propagation as well as damage states in the presence of noise. The peak amplitude coefficient and AR coefficient perform better (AUC = 1, 0.989) to classify damage from the undamaged state for the sensor pair 01–04 in this structure. This result is not unexpected because features CC and CWT coefficient vales fluctuate suddenly, which miss-classified the damage state from undamaged sate. For the proposed feature, the performance varies for different threshold values. Hence, even though there may be the best feature for a particular transducer pair and a specific threshold, so it may be suitable to use the information from all features from different sensors to reach better localization or detection of crack/changes. The output of Table 2 shows that the peak amplitude coefficient feature extracted from the ultrasonic signal performs better (AUC = 1) compared to all the NDT methods in this benchmark structure. It can be observed that the ultrasonic sensor detects crack (damage) located between the pair of sensors earlier than other NDT methods.

### 3.6. Crack Opening Displacement

**Figure 22:**
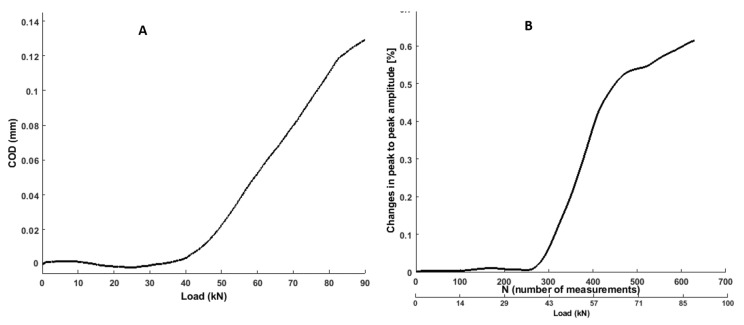
Load-COD (**A**) vs. load-changes in peak to peak amplitude (**B**) response of a RC benchmark structure.

The crack opening displacement (COD) of reference benchmark structure was determined through the DIC method. To determine the crack opening displacements (COD), the displacement fields were used [50]. COD values were achieved by considering the discontinuity of two point (middle of the beam) by following the crack path (central crack), the transform of the dissipated energy and the crack opening along the crack path was measured (anticipating the crack opening width vs. depth line to zero). As peak to peak amplitude perform better (AUC = 1) than other ultrasonic features, therefore it was presented to link with COD. Figure 22 shows a comparison between the changes in peak to peak amplitude (absolute value) from ultrasonic feature and those measured with DIC method. One can see that COD was well matched with ultrasonic feature.

### 3.7. Discussion

The ultrasonic transducers are located on top and bottom and inside the concrete element subjected to a tensile force. The post-processing of the signals in time and frequency domains as well as by the continuous wavelet transform is a base for early crack detection algorithm developed in this study. The characteristics of each stage are described below:

- At the beginning of the test, all the features of the test specimen were weak, displaying minimal changes (energy release). At this stage, the specimen was under a confining pressure and axial loading stress on the top (*N* = 240). The specimen was experiencing elastic deformation.
- The sudden decrease of change/damage index from all the features could be observed before the appearance of the first vertical cracks observed by DIC.
- In the second stage, the rate of changes in all the features remained at a high level. Due to the rapid expansion of internal cracks in the concrete, the reinforced concrete produced large deformation. The changes in the ultrasonic features were extremely intensive. Therefore, the rate of the structural changes reached its peak (energy was released from multiple cracks), even when the load was decrease to 20 kN (after the test) but the changes remain same (*N* = 800, after 170 kN). This can be used as a parameter for final decision about structural condition.
- As it can be noticed from Table 2, all the sensors detected the crack properly and with a very high sensitivity. This is because the cracks appear in the middle of the tested beam where most of the sensors were located.
- The declaration of the structural status (“damaged” and “undamaged”) is very important, therefore proposed information-based fusion using voting index provide more accurate results (e.g., AR feature provided misleading damage status when the beam was undamaged stage (*N* = 50, after 7 kN)).
- At this stage of research, the study has been limited to diagnostics of reinforced concrete reference structure based on four ultrasonic transducers. Such configuration allows detecting damage within the path of propagating waves without the possibility of precise damage localization. It is important to note that ultrasonic sensors and the related features are the most sensitive to initiating and propagating cracks among the measurement techniques considered in this study.
- The two pairs of ultrasonic transducers can reveal the concrete damage process of constituent and interfaces in different ways. The signal-level fusion approach to combine the information coming from both pair of sensors should be integrated to precisely and accurately predict the concrete damage evolution. This goal is the next research step of the authors.

## 4. Conclusions

The presented study was aimed to evaluate various structural testing techniques to detect early cracking behavior in RC structures. For this purpose, various testing techniques and processing algorithms were used. Apart from external sensors, the primary attention was paid to embedded ultrasonic sensors. The ability of diffuse ultrasonic technique in monitoring the cracking behavior of the tested beam specimens was verified. Similar results were observed when the load-crack opening curves obtained from ultrasonic features and traditional sensors were compared. For the detection of changes in the RC benchmark structure, the most sensitive NDT method is the diffusing ultrasonic sensors. From the ultrasonic features, the formation of microcracks inside the beam is detected and localized. This result clearly implied the great ability of ultrasonic features to detect the crack opening and crack propagation. The peak to peak amplitude and AR coefficient indicator is interesting since it both continuously evolves and follows well the three different phases which describe the failure mechanisms that are the microcracks initiation, the propagation of cracks, and the final failure. Thanks to the use of the CWT coefficient feature, the damage could even be detected before it reaches the surface. The evaluation of the ultrasonic signal attenuation leads to the early crack detection, even before this crosses the direct wave path.

The DIC, on the other hand, detects the smallest deformations of the surface caused by the bending of the beam. Crack developments through the section depth were also monitored by DIC, and it was found that the width of the crack sustained for a certain stage of loading. When multiple cracking behavior is observed, the advantage of the DIC method over other measurement techniques was noticed, since cracks appear on concrete face parallel to the load application lead to registering of increased values. The exact cracking locations cannot be predicted with other methods. The study has furthermore shown that the use of features and feature-based fusion improves the overall decision based on the detection capability of a multisensor system located in different places of the structure.

Finally, it can be concluded that ultrasonic measurements have the potential to be used as an alternative to more traditional sensors, and, offer significant advantages over traditional measurement techniques because it can provide full-field surface strain measurements, as well as be advantageous in determining crack opening and propagation. Most importantly, the ultrasonic feature can monitor even tiny strains/cracks during loading. Therefore, this part of our work is in progress. The effect of signal-level fusion on all transducer pairs to find localization will be investigated. Also, the effectiveness of ultrasonic sensors for long term monitoring in the real structure will be in the focus of further studies.

## Figures and Tables

**Table 1:** Description of the features.

Feature	Description	Equation
Peak Amplitude	Distinctive peak to peak amplitude	$P_{a}=\frac{\left[PA_{measured}-PA_{reference}\right]}{PA_{reference}}$
AR	Distinctive of AR amplitude	$\epsilon \left(t\right)=x\left(t\right)-\sum_{i=1}^{n}\alpha_{i}\bar{x}\left(t-i\right)+e_{m}$
CC	Distinctive of the waveform changes	$D_{cc}=1-\rho_{xy}$
CWT	Differential energy in frequency domain using wavelet transform	$CWT_{c}=\sqrt{\frac{\sum_{j=1}^{n}{\left(CWT_{e}{,}_{j}-CWT_{e}{,}_{1}\right)}^{2}}{\sum_{j=1}^{n}(CWT_{e}{,}_{1}{)}^{2}}}$
STFT	Differential energy in frequency domain	$STFT_{c}=\sqrt{\frac{\sum_{j=1}^{n}{\left(STFT_{e}{,}_{j}-STFT_{e}{,}_{1}\right)}^{2}}{\sum_{j=1}^{n}(STFT_{e}{,}_{1}{)}^{2}}}$

**Table 2:** Overall results for different NDT techniques.

NDT Methods	AUC
Ultrasonic	1
LVDT	0.994
DIC (deflection)	0.992
Strain gauge	0.985

## Abbreviations

The following abbreviations are used in this manuscript:

RC	Reinforced concrete
BAM	Bundesanstalt für Materialforschung und -prüfung
NDT	Non-Destructive Testing
CWI	Coda wave interferometry
AR	Autoregressive model
CC	Decorrelation coefficient
CWT	Continuous wavelet transform
STFT	Short-time Fourier transform
UPV	Ultrasonic pulse velocity
AE	Acoustic emission
DIC	Digital image correlation and tracking
LVDT	Linear variable differential transducer
ROC	Receiver operating characteristic
